# DGKZ promotes TGFβ signaling pathway and metastasis in triple-negative breast cancer by suppressing lipid raft-dependent endocytosis of TGFβR2

**DOI:** 10.1038/s41419-022-04537-x

**Published:** 2022-02-03

**Authors:** Yuanyuan Zhao, Hefen Sun, Xuan Li, Qiqi Liu, Yang Liu, Yifeng Hou, Wei Jin

**Affiliations:** 1 Department of Breast Surgery, Key Laboratory of Breast Cancer in Shanghai, Fudan University Shanghai Cancer Center, Shanghai, 200032 China; 2 Department of Oncology, Shanghai Medical College, Fudan University, Shanghai, 200032 China

**Keywords:** Breast cancer, Cell migration

## Abstract

Diacylglycerol kinase ζ (DGKZ) is a diacylglycerol kinase that metabolizes diacylglycerol to yield phosphatidic acid, and its function in breast cancer progression remains unclear. In this study, via screening of a CRISPR-Cas9 knockout library containing lipid metabolic genes, DGKZ was identified as a potential prometastatic gene. We first confirmed that high DGKZ expression correlated with tumor progression and poor prognosis in patients. Next, knockout of DGKZ in triple-negative breast cancer cell lines were found to significantly inhibit metastatic behaviors in vitro and in vivo, whereas its overexpression increased the metastatic potential of cell lines. Mechanistic studies based on RNA sequencing and bioinformatic analysis indicated that DGKZ might regulate cell metastasis by promoting epithelial–mesenchymal transition via the transforming growth factor β (TGFβ) signaling pathway. Furthermore, we found that overexpression of DGKZ activated the TGFβ/TGFβR2/Smad3 signaling pathway by inhibiting the degradation of TGFβR2 through suppression of caveolin/lipid raft-dependent endocytosis. Moreover, the caveolin/lipid raft-dependent endocytosis of TGFβR2 was regulated by the metabolite phosphatidic acid, which might alter TGFβR2 partitioning in lipid rafts and nonlipid rafts by affecting the fluidity of the plasma membrane. These findings suggested that DGKZ is a novel promoter of metastasis and that it could be a potential prognostic indicator in patients with triple-negative breast cancer.

## Introduction

Breast cancer is the most common malignant tumor that endangers women’s health worldwide, and it has attracted increasing attention due to its high morbidity and mortality [1]. Breast cancer with negative estrogen receptor (ER), negative progesterone receptor (PR), and negative human epidermal growth factor receptor 2 (HER2) expression is pathologically defined as triple-negative breast cancer (TNBC) [2]. It accounts for 12–17% of all breast cancers and is also called refractory breast cancer because of its unclear therapeutic target, high malignancy, high risk of early recurrence and metastasis, and poor prognosis [3]. An understanding of the mechanisms and the identification of biomarkers that drive metastasis may help improve survival [4]. The lack of targeted therapies and the poor prognosis of patients with TNBC have fostered a major effort to discover actionable molecular targets to treat patients with these tumors [5–7].

Metabolic reprogramming plays an important role in the tumorigenesis and progression of cancers. There are a large number of studies on the correlations of glycolysis and glutamine metabolism with metastasis [8–10], but whether lipid metabolism can affect the malignant progression of breast cancer tumors needs to be explored. It is known that a variety of candidates involved in lipid metabolism may also be involved in tumorigenesis and progression [11, 12]. For example, the lipid metabolism-related gene ACSS3 was found to reduced lipid droplet deposition by regulating the stability of the lipid droplet coat protein PLIN3 and to inhibited prostate cancer progression and resistance to new endocrine therapies by reducing intratumoral lipid accumulation [13]. A recent study also showed that long-chain fatty-acid CoA ligase 1 (ACSL1) expression can convert the lipid profile of nonmetastatic ovarian cancer cells to a profile similar to that of highly metastatic ovarian cancer cells and make them highly aggressive. ACSL1 can also activate the AMP-activated protein kinase and Src pathways via protein myristoylation, regulate fatty acid beta-oxidation, and finally enhance cancer metastasis [14]. However, the role of most lipid molecules and enzymes in the molecular mechanism of triple-negative breast cancer metastasis remains unclear.

We preliminarily retrieved Gene Cards and NCBI datasets to search for lipid metabolism-related gene arrays, utilized a CRISPR-Cas9 knockout library to screen for functional genes, and finally enriched a number of candidate genes that may affect TNBC metastasis. In our previous study, we found that NAD(P) dependent steroid dehydrogenase-like (NSDHL), a cholesterol metabolic enzyme, was a metastatic driver in TNBC [15]. We also found that diacylglycerol kinase ζ (DGKZ), one of the other candidate genes, might be associated with metastasis.

The protein encoded by the DGKZ gene belongs to the eukaryotic diacylglycerol kinases (DGK) family, which is involved in catalyzing the conversion of diacylglycerol (DAG) to phosphatidic acid (PA) [16, 17]. DAG and PA are members of a class of lipid molecules with unique biological functions that can work as metabolic intermediates, basic components of biofilms, or second messengers [18]. Currently, the role of DGKZ in TNBC metastasis has not been identified. This study used bioinformatics and molecular biology approaches to explore the regulation of potential molecular targets mediating for TNBC metastasis and showed that DGKZ is a promoter of TNBC metastasis for the first time.

## Results

### DGKZ is upregulated in triple-negative breast cancer cells

**Fig. 1:**
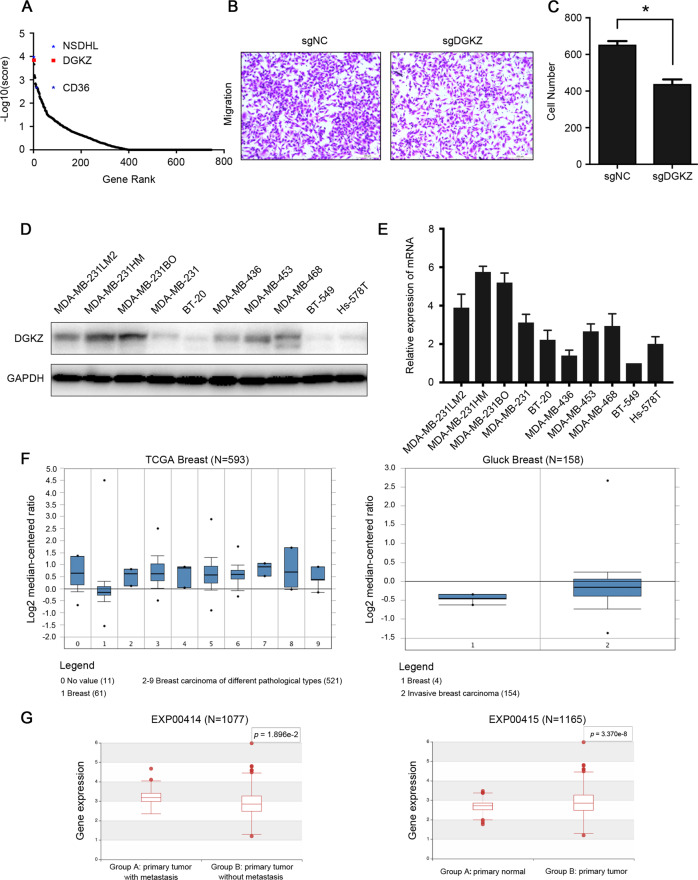
DGKZ is upregulated in highly metastatic TNBC cells. **A** Explicit oncogenes NSDHL and CD36 were enriched in the negative selected gene sequencing, and DGKZ was located in the front place in the sequencing. **B** The change in migration efficiency was analyzed by transwell assay in vitro after DGKZ was knocked out in the negative selected gene sequencing. **C** The histogram of the number of migrated cells in section **B**. **D**–**E** The protein and mRNA expression of DGKZ in TNBC cell lines (protein expression of DGKZ in MDA-MB-231 and mRNA expression of DGKZ in BT-549 cell as the reference). **F** Analysis of DGKZ expression in multiple subtypes of human breast cancer in the Oncomine database. **G** Analysis of DGKZ expression in primary normal breast tissues, primary tumor tissues, and primary tumor tissues with/without metastasis in the Human Cancer Metastasis Database (HCMDB). The data represent the mean ± SD of three independent experiments. Bars, ±SD; *p < 0.05.

In our previous study, we analyzed genes related to lipid metabolism with the NCBI and GeneCards databases and constructed the CRISPR-Cas9 gene knockout library. Through in vivo screening, second-generation sequencing, and bioinformatics analysis, some candidate genes that may affect the metastasis of TNBC were found. The MAGeCK algorithm was used to calculate and sequence the positive selected genes (potential tumor-suppressing genes) and negative selected genes (potential tumor-promoting genes). Among the top 15 negative selected genes, we found some explicit oncogenes, such as NSDHL and CD36 (Fig. 1A), suggesting that the screening model was accurate and reliable. Then, we selected some less-studied genes and tested them by transwell assay in vitro, finding that the migration ability of MDA-MB-231LM2 cells was significantly reduced after we inhibited the expression of DGKZ (ranked frontal among the negative selected genes) (Fig. 1B, C).

A panel of TNBC cell lines was also evaluated to examine the expression of DGKZ, and we found that the mRNA and protein expression levels of DGKZ were relatively low in TNBC cell lines with weak metastatic potential (Hs-578T and BT-549 cells), while the highly metastatic cell lines (MDA-MB-231HM and MDA-MB-231LM2) exhibited higher DGKZ expression (Fig. 1D, E). In addition, analyses of the Oncomine database and Human Cancer Metastasis Database (HCMDB) revealed that DGKZ was upregulated in breast carcinoma compared with normal breast tissues. The HCMDB results also indicated that DGKZ expression was significantly increased in the primary tumor tissues with metastasis compared with those without metastasis (Fig. 1F, G).

In addition, we evaluated the clinical prognostic value of DGKZ using Kaplan–Meier plotter. Although no significant correlation was found between prognosis and DGKZ expression in breast cancer patients, survival analysis showed that the overall survival (OS) of lung cancer, gastric cancer and renal clear cell cancer patients negatively correlated with DGKZ expression (Supplementary Fig. 1). Taken together, these data suggested that DGKZ might play an oncogenic role in multiple cancer types, making it worthy of further investigation.

### Loss of DGKZ inhibits TNBC migration and metastasis in vitro and in vivo

**Fig. 2:**
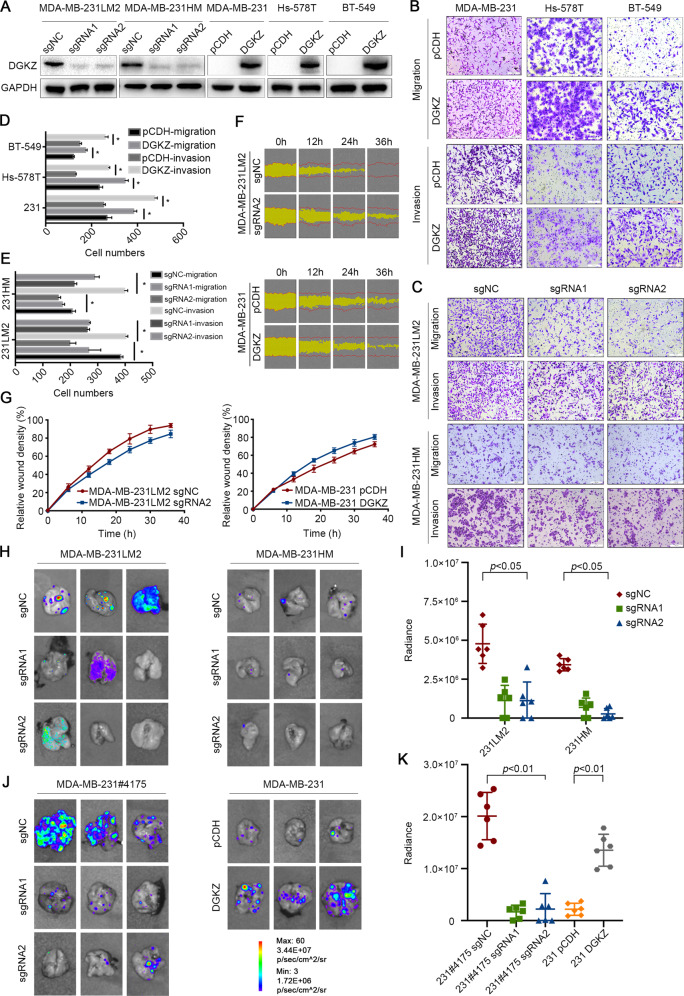
Loss of DGKZ inhibits TNBC migration and metastasis in vitro. **A** DGKZ was stably knocked out in highly metastatic MDA-MB-231LM2 and MDA-MB-231HM cells using two sgRNAs, and it was overexpressed in MDA-MB-231, Hs-578T, and BT-549 cells. **B**–**E** The migration and invasion abilities of the above cell lines were evaluated by transwell assays in vitro. The right panel shows photos of representative fields (100× magnification), and the left panel shows the histograms of the results. **B**–**D** for MDA-MB-231, Hs-578T, and BT-549 cells and **C**–**E** for MDA-MB-231LM2 and MDA-MB-231HM cells. Statistical analysis was performed using Student’s *t*-test (*n* = 3). **F**, **G** Effects of DGKZ on the migration of MDA-MB-231 and MDA-MB-231LM2 cells based on the wound-healing assay. The red lines indicate the initial scratch wound location, and the yellow area demonstrates scratch wound closure. Images were acquired every 6 h after wounding. **H** and **J** Bioluminescent imaging of three representative mouse lungs in each group at week 6–8 after injection with DGKZ-overexpressing MDA-MB-231 or DGKZ-knockout MDA-MB-231LM2, MDA-MB-231HM, and MDA-MB-231#4175 cells. **I** and **K** Maximum bioluminescence imaging signals of each mouse’s lung. The data represent the mean ± SD of three independent experiments. Bars, ±SD; *p < 0.05.

To further investigate the role of DGKZ in tumor metastasis, we constructed cells with stable overexpression and knockout of DGKZ in several TNBC cell lines. Western blot analysis demonstrated that DGKZ was obviously overexpressed in Hs-578T, MDA-MB-231, and BT-549 cells. However, DGKZ expression was markedly reduced in MDA-MB-231LM2 and MDA-MB-231HM cells after CRISPR-Cas9 gene editing (Fig. 2A). Then, we evaluated the effect of DGKZ on the malignant phenotype of TNBC cells with a transwell assay. The results showed that overexpression of DGKZ significantly promoted migration and invasion in Hs-578T, MDA-MB-231, and BT-549 cells (Fig. 2B, D), while knockout of DGKZ impaired the migration and invasion potential of MDA-MB-231LM2 and MDA-MB-231HM cells (Fig. 2C, E). Using a wound-healing assay, we also found that DGKZ overexpression elevated migration in MDA-MB-231 cells, while DGKZ depletion suppressed the migration ability of MDA-MB-231LM2 cells (Fig. 2F, G). These results confirmed that DGKZ promoted the metastatic properties of TNBC cells in vitro.

**Fig. 3:**
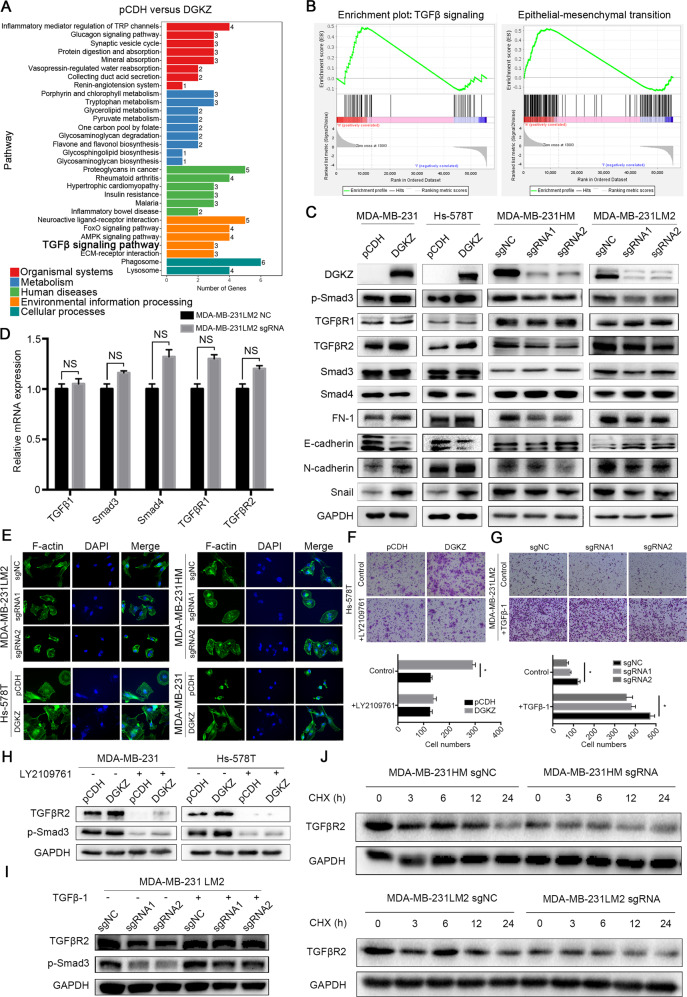
DGKZ promotes epithelial–mesenchymal transition via the transforming growth factor β signaling pathway. **A** Signaling pathways enriched based on the KEGG pathway analysis with the RNA sequencing data from DGKZ-upregulated BT-549 cells. **B** GSEA indicated that epithelial–mesenchymal transition and the transforming growth factor β signaling pathway were upregulated in DGKZ-overexpressing cells. **C** Protein levels of the EMT markers FN-1, E-cadherin, N-cadherin, and Snail and the TGFβ pathway markers p-Smad3, TGFβR1, TGFβR2, Smad3, and Smad4 in cell lines after DGKZ overexpression or downregulation based on western blot. **D** RNA expression of the TGFβ pathway markers TGFβ1, TGFβR1, TGFβR2, Smad3, and Smad4 in MDA-MB-231LM2 cells after DGKZ was downregulated based on real-time PCR. **E** F-actin staining of DGKZ-overexpressing MDA-MB-231 and Hs-578T cells or DGKZ-knockout MDA-MB-231LM2 and MDA-MB-231HM cells based on immunofluorescence. **F** Transwell assay of Hs-578T cells treated with the TGFβ receptor inhibitor LY2109761 (10 μM) for 48 h. **G** Transwell assay of MDA-MB-231LM2 cells treated with the pathway agonist TGFβ-1 (10 ng/l) for 48 h. **H** Western blot analysis of MDA-MB-231 and Hs-578T cells overexpressing DGKZ treated with LY2109761 (10 μM) for 48 h. **I** Western blot analysis of MDA-MB-231LM2 cells with stable knockout of DGKZ treated with TGFβ-1 (10 ng/l) for 48 h. **J** Western blot analysis of MDA-MB-231LM2 and MDA-MB-231HM cells downregulating DGKZ treated with cycloheximide (50 µM) for 24 h. The data represent the mean ± SD of three independent experiments. NS, no significance; Bars, ±SD; *p < 0.05.

Next, to assess whether DGKZ affects metastasis in vivo, we established four groups of mouse models. DGKZ knockout and overexpressing cells were labeled with a retroviral construct expressing a GFP/luciferase fusion protein [19]. The mice were then monitored by noninvasive bioluminescence imaging (BLI) 4–6 weeks after intravenous injection of cells into BALB/c nude mice or orthotopic injection into NOD-SCID mice. Our results demonstrated that downregulation of DGKZ significantly decreased lung metastasis in mice injected with MDA-MB-231LM2, MDA-MB-231HM, and MDA-MB-231#4175 cells. In addition, DGKZ upregulation elevated lung metastasis in mice injected with MDA-MB-231 cells (Fig. 3H–K and supplementary Fig. 2). These findings confirmed our hypothesis that high DGKZ expression promotes TNBC cell metastasis in vitro and in vivo.

Besides, we also found that high DGKZ expression promoted TNBC cell proliferation in vitro and in vivo (supplementary Fig. 3).

### DGKZ promotes epithelial–mesenchymal transition via the transforming growth factor β signaling pathway

To uncover the mechanism underlying DGKZ-mediated metastasis, we performed RNA sequencing combined with bioinformatic analysis in DGKZ-overexpressing BT-549 cells. KEGG pathway analysis revealed that the transforming growth factor β (TGFβ) signaling pathway was activated in DGKZ-overexpressing cells (Fig. 3A). Moreover, GSEA showed that overexpression of DGKZ significantly elevated epithelial–mesenchymal transition (EMT) and the TGFβ pathway (Fig. 3B). Then we performed western blotting to confirm whether DGKZ affects the TGFβ pathway and EMT, and the results showed that depletion of DGKZ significantly downregulated the protein levels of p-Smad3 and TGFβR2 in the TGFβ pathway. In addition, the expression of Smad3, Smad4, and TGFβR1 was not significantly changed with DGKZ overexpression. Meanwhile, low expression of DGKZ significantly decreased the expression of the EMT markers N-cadherin (N-CAD), fibronectin 1 (FN-1), and Snail but upregulated E-cadherin (E-CAD). DGKZ overexpression produced the opposite effect (Fig. 3C). Furthermore, the mRNA expression of TGFβ1, Smad3, Smad4, TGFβR1, and TGFβR2 was not significantly altered with the deletion of DGKZ (Fig. 3D).

Cell invasion is the crucial primary step in cancer cell metastasis. Cells escaping from the primary cancer site require increased motility and migration, and EMT might be the underlying mechanism for this process. To determine whether DGKZ regulates the cytoskeleton, we observed alterations in the cytoskeleton by performing F-actin staining. MDA-MB-231 and Hs-578T cells with elevated DGKZ expression exhibited significantly elongated cytoskeletons with more pseudopodia, indicative of EMT. In contrast, in MDA-MB-231LM2 and MDA-MB-231HM cells, the downregulation of DGKZ notably suppressed the length and number of pseudopodia (Fig. 3E). These results suggested that DGKZ could regulate cell migration via cytoskeletal rearrangements and EMT.

To further verify our findings, we detected the protein levels of p-Smad3 and TGFβR2 and observed that they declined significantly after the addition of the TGFβ receptor inhibitor LY2109761 to MDA-MB-231 and HS-578T cells overexpressing DGKZ. Moreover, transwell migration assays suggested that the addition of LY2109761 could neutralize the enhanced tumor migration efficiency induced by DGKZ overexpression (Fig. 3F, H). On the other hand, the addition of the signaling agonist TGFβ−1 to DGKZ-depleted 231LM2 cells had the opposite effect (Fig. 3G, I).

These results suggested that DGKZ could activate the TGFβ-mediated EMT pathway to promote tumor metastasis.

### Overexpression of DGKZ inhibits the degradation of TGFβR2 by suppressing lipid raft-mediated TGFβR2 endocytosis

Our previous results have shown that DGKZ upregulated the protein expression of TGFβR2 but did not alter its mRNA expression; therefore, we wondered whether DGKZ affected the degradation of TGFβR2. The results showed that TGFβR2 had a faster degradation rate in DGKZ-depleted cells than in control cells when they were treated with cycloheximide (a protein synthesis inhibitor, CHX) (Fig. 3J). To further identify whether DGKZ inhibits the degradation of TGFβR2 by directly affecting the ubiquitin-proteasome system (UPS) or lysosomal degradation, cells were treated with MG132 (a ubiquitin–proteasome inhibitor), NH4CL (a lysosomal pathway inhibitor), and bafilomycin A1 (a lysosomal pathway inhibitor, BAF1), and there was no significant difference between the treatment group and the control group (Supplementary Fig. 5).

**Fig. 4:**
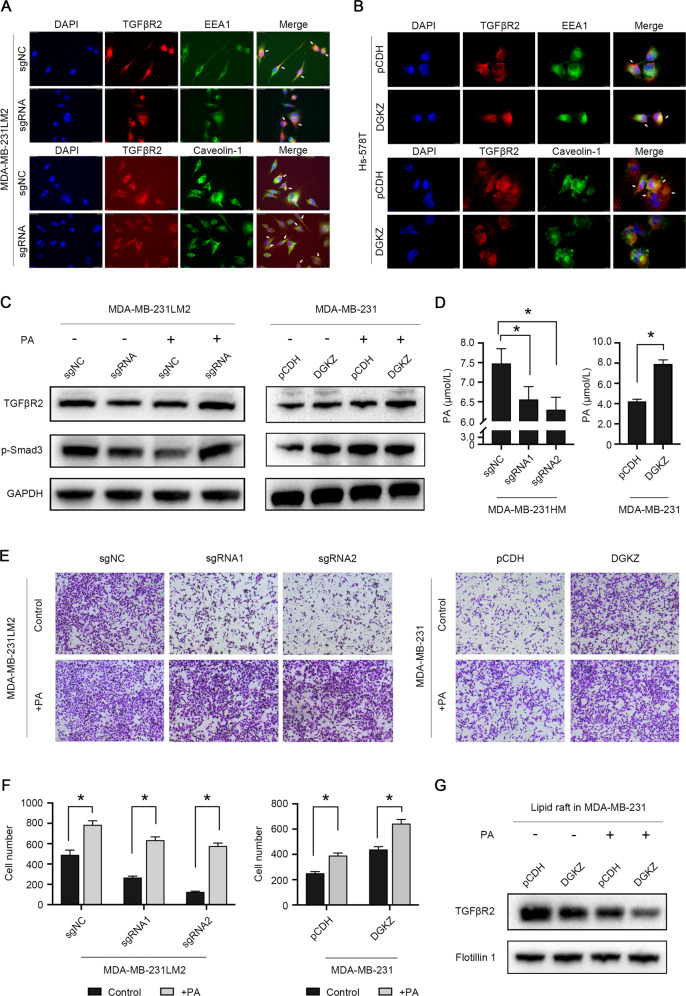
Overexpression of DGKZ activates the TGFβ signaling pathway by inhibiting the degradation of TGFβR2. **A** Effect of the knockout of DGKZ on the colocalization of TGFβR2 (red) and EEA1 (green) or Caveolin-1 (green). **B** Effect of the overexpression of DGKZ on the colocalization of TGFβR2 (red) and EEA1 (green) or Caveolin-1 (green). The arrows demonstrate the colocalization of TGFβR2 with EEA1 or Caveolin-1. At least six visual fields were randomly selected from each group for photographing and recording, and the magnification was 10 × 63. **C** Western blot analysis of MDA-MB-231LM2 cells with DGKZ-knockout and MDA-MB-231 cells overexpressing DGKZ treated with PA (10 μM) for 72 h. **D** ELISA of MDA-MB-231 cells overexpressing DGKZ and MDA-MB-231HM cells with stable knockout of DGKZ to evaluate the intracellular level of phosphatidic acid. **E**, **F** Transwell assay of MDA-MB-231LM2 and MDA-MB-231 cells treated with PA (10 μM) for 72 h. **G** Western blot analysis of the DGKZ-overexpressing 231 cells treated with PA (10 μM) for 72 h and isolated the lipid rafts on the plasma membrane. The data represent the mean ± SD of three independent experiments. Bars, ±SD; *p < 0.05.

The above results demonstrated that DGKZ suppresses the degradation of TGFβR2 independent of the canonical UPS or lysosomal degradation. It was reported that the internalization of various proteins occurs through the clathrin- and caveolin-positive lipid-raft endocytic pathways, including TGFβRs [20]. Therefore, to further investigate whether DGKZ alters the internalization of TGFRβ2, we performed an immunofluorescence assay to explore the colocalization of TGFβR2 with the primary endosome marker EEA1 and the lipid raft marker Caveolin-1. Our results demonstrated that the colocalization of TGFβR2 with the primary endosome marker EEA1 decreased but its colocalization with the lipid raft marker Caveolin-1 increased in DGKZ-knockout 231LM2 cells (Fig. 4A). In contrast, the colocalization of TGFβR2 with EEA1 increased and the colocalization of TGFβR2 with Caveolin-1 decreased in the DGKZ-overexpressing Hs-578T cells (Fig. 4B). These results indicated that DGKZ decreased the localization of TGFβR2 in lipid rafts and suppressed lipid raft-caveolar endocytosis.

Next, we verified whether DGKZ affected the partitioning of TGFβ2 in the cell membrane by altering its metabolic product PA. We first performed ELISA (enzyme-linked immunosorbent assay) to detect whether DGKZ altered the PA level. The results showed that the intracellular level of PA in MDA-MB-231HM cells was decreased after the deletion of DGKZ. However, PA was significantly increased after overexpression of DGKZ in MDA-MB-231 cells (Fig. 4D). To further confirm that PA might affect the degradation of TGFβR2 as well as the cell migration ability, we incubated DGKZ-overexpressing MDA-MB-231 cells and DGKZ-depleted MDA-MB-231LM2 cells with PA (10 μM for 72 h). Western blot results showed that the activation or inhibition of the TGFβ signaling pathway was further enhanced or restored after the addition of PA (Fig. 4C), and the migration ability of cells was also enhanced (Fig. 4E, F). In addition, we analyzed the protein level of TGFβR2 in lipid rafts on the plasma membrane in DGKZ-overexpressing 231 cells treated with PA after lipid raft isolation. Western blot analysis revealed that TGFβR2 in the lipid rafts decreased in the DGKZ-overexpressing cells and that the retention of TGFβR2 in the lipid rafts decreased more significantly after the addition of PA (Fig. 4G).

These results indicated that DGKZ might change the fluidity of the plasma membrane through the metabolite PA, which leads to the redistribution of TGFβR2 in the plasma membrane from the lipid raft area to the nonlipid raft area, suppressing lipid raft-mediated TGFβR2 endocytosis and activating the TGFβ signaling pathway.

### High DGKZ expression correlates with poor prognosis

**Fig. 5:**
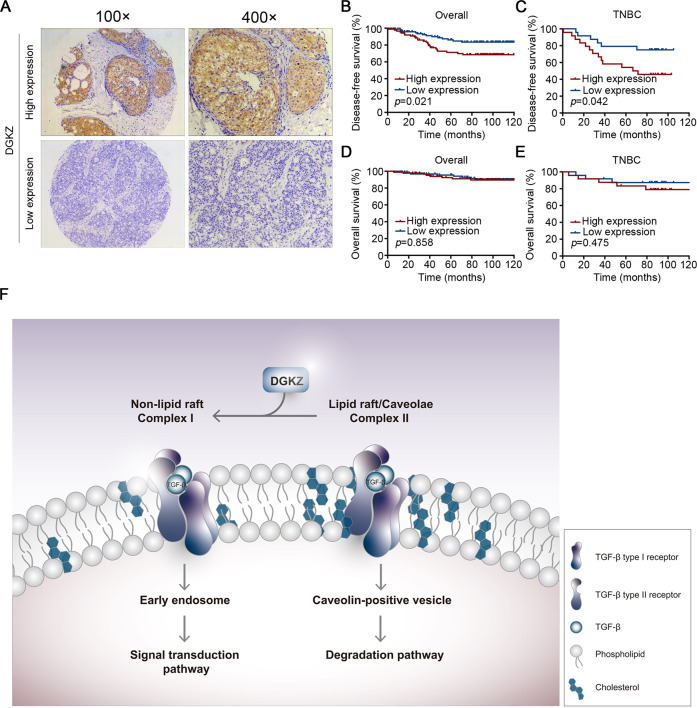
High DGKZ expression correlates with poor prognosis. **A** Immunohistochemical analysis of the expression of DGKZ in TMAs. **B**, **C** The correlation of the expression of DGKZ with disease-free survival (DFS) in all patients and TNBC subtypes. **D, E** The correlation of the expression of DGKZ with overall survival (OS) in all patients and TNBC subtype. **F** A novel model for the regulation of the TGFβ signaling pathway by DGKZ.

We found that DGKZ was a metastasis driver gene in TNBC cell lines. To further observe the clinical relevance of the above findings in human breast cancer patients, we detected the expression of DGKZ in TMAs containing 200 breast tumor specimens by performing IHC analysis (Fig. 5A). DGKZ was highly expressed in 93 cases of breast cancer and expressed at low levels in 103 cases. The correlation between DGKZ expression and clinicopathological characteristics was further analyzed. The results suggested that cases with negative progesterone receptor (PR) expression accounted for 59.1% of the high DGKZ expression patients, while cases with positive PR expression accounted for 40.9%, and the difference was statistically significant (*p* = 0.031). Moreover, age, grade, tumor size, lymph node status, ER status, and HER2 status had no significant correlation with DGKZ expression (Table 1).

**Table 1:** The correlation between DGKZ expression and clinicopathological characteristics.

Variables		Low expression		High expression		*p*-value
		*n* = 103	%	*n* = 93	%	
Age (years)	<49	52	50.5	44	47.3	0.657
	≥50	51	49.5	49	52.7	–
Grade	I	1	1.0	2	2.2	0.739
	II	71	68.9	66	71.0	–
	III	31	30.1	25	26.9	–
T stage	1	51	49.5	46	49.5	0.705
	2	48	46.6	41	44.1	–
	3	4	3.9	6	6.5	–
Lymph nodes	Negative	42	40.8	37	39.8	0.888
	Positive	61	59.2	56	60.2	–
Hormone status	ER^1^ positive	44	42.7	50	53.8	0.122
	PR^2^ positive	58	56.3	38	40.9	0.031
Her2^3^ status	Negative	61	59.2	54	58.1	0.869
	Positive	42	40.8	39	41.9	–

*ER*^1^ Estrogen receptor, *PR*^2^ Progesterone receptor, *Her2*^3^ Epidermal growth factor receptor 2

Of note, high DGKZ expression was significantly correlated with poor disease-free survival (DFS) in all patients (*p* = 0.021) and in the TNBC subtype (*p* = 0.042). However, there was no significant correlation between DGKZ expression and overall survival (OS) in all cases or in the TNBC subtype (Fig. 5B–E).

Finally, univariate analysis was performed on 196 patients using a Cox regression model to explore the effects of different variables on DFS, indicating that tumor size, lymph node status, hormone receptor status, and DGKZ expression level were correlated with disease-free survival. Additionally, multivariate analysis revealed that high expression of DGKZ was an independent risk factor for breast cancer recurrence and metastasis (Table 2).

**Table 2:** The univariate analysis and multivariate analysis between clinicopathological characteristics and DFS.

Variables		Univariate analysis		Multivariate analysis	
		HR (95% CI^1^)	*p*-value	HR (95% CI)	*p*-value
Age (years)	<49	Reference	–	Reference	–
	≥50	1.010 (0.979-1.041)	0.542	1.279 (0.652-2.507)	0.474
Grade	I	Reference	–	Reference	–
	II	0.922 (0.423-1.902)	0.974	0.912 (0.456-1.856)	0.833
	III	0.931 (0.435-1.925)	0.916	0.947 (0.461-1.918)	0.921
T stage	1	Reference	–	Reference	–
	2	1.321 (0.687-2.541)	0.405	0.971 (0.477-1.975)	0.935
	3	3.258 (0.948-11.205)	0.041	2.463 (0.673-9.010)	0.173
Lymph nodes	Negative	Reference	–	Reference	–
	Positive	2.981 (1.783-6.953)	0.006	2.482 (1.263-4.880)	0.008
ER^2^	Negative	Reference	–	Reference	–
	Positive	0.412 (0.205-0.827)	0.013	0.401 (0.125-1.289)	0.125
PR^3^	Negative	Reference	–	Reference	–
	Positive	0.480 (0.239-0.964)	0.039	0.944 (0.297-3.001)	0.922
Her2^4^	Negative	Reference	–	Reference	–
	Positive	0.840 (0.444-1.590)	0.592	0.701 (0.358-1.375)	0.302
DGKZ	Low	Reference	–	Reference	–
	High	2.125 (1.104-4.089)	0.024	2.413 (1.225-4.756)	0.011

*CI*^1^ Confidence interval; *ER*^2^ Estrogen receptor; *PR*^3^: Progesterone receptor; *Her2*^4^ Epidermal growth factor receptor 2

These findings revealed the utility of DGKZ for prognostic stratification of patients with breast cancer and confirmed our hypothesis that DGKZ promotes the metastasis of human triple-negative breast cancer by activating SMAD-dependent TGFβ signaling.

## Discussion

The protein encoded by the DGKZ gene belongs to the eukaryotic diacylglycerol kinase (DGK) family, and several members of this family have been found to participate in tumorigenesis, progression, and drug resistance. For example, DGKA is upregulated in melanoma, hepatocarcinoma and glioblastoma, where it contributes to the acquisition of tumor metastatic traits [21], and induces cisplatin and anti-PD-1 antibody resistance via crosstalk with kinase and transcription networks [22, 23]. In addition, several studies have revealed that DGKZ functions as a promotor in human glioma [24], colorectal cancer [25, 26], and cervical cancer [27], but its role in breast cancer remains unclear. By using Oncomine expression analysis, we recognized that DGKZ underwent a copy number elevation in tissues of invasive breast cancer, suggesting that the potential importance of DGKZ in oncogenesis and progression of breast cancer. On the basis of data from the HCMDB, the expression level of DGKZ is much higher in primary breast tumors or primary tumors with metastasis than in normal breast tissue or primary tumors without metastasis. Moreover, high DGKZ expression is closely associated with lung metastasis in mouse breast cancer models. TMAs of samples from breast cancer patients revealed that high DGKZ expression correlated with poor prognosis. Our results suggest that further studies should be established to find the potential clinical utility of DGKZ as a prognostic biomarker for aggressive breast carcinoma.

Previous studies have demonstrated that DGKZ promotes proliferation and invasion through multiple mechanisms. For instance, elevated DGKZ expression contributes to increased Rho GTPase activation and the enhanced motility of metastatic cancer cells [26]. Furthermore, DGKZ activation downstream of mTORC2 in rapamycin-resistant colorectal tumors helps to maintain the DAG/PA balance necessary for cell survival, and DGKZ suppression potentiates the growth-inhibitory effect of rapamycin [25]. A recent study showed that differentially expressed proteins induced by silencing of DGKZ in cervical cancer were mostly enriched in autophagy or mitophagy, indicating that the functions of DGKZ in cell proliferation and tumor growth may be associated with autophagy or mitophagy. Further analysis showed that the PI3K-AKT and TAK1-NF-κB signaling pathways were prominently inhibited in SiHa cells transfected with sh-DGKZ [27].

Our study showed that the phenotype of TNBC cells with DGKZ upregulation was consistent with the induction of a mesenchymal transdifferentiation program. Overexpression of DGKZ significantly reduced the expression of E-cadherin, while increasing the expression of N-cadherin and FN-1, and the cytoskeletal morphology of the cells had significant changes, indicating that DGKZ participated in the induction of EMT in triple-negative breast cancer cells. Then we confirmed that DGKZ partially regulated TNBC progression via the TGFβ-SMAD signaling pathway.

Tumor cells normally secrete abundant TGFβ, which is the most potent inducer of EMT in epithelial cancers and promotes cell invasion and metastasis [28, 29]. TGFβ exerts its effects through the activation of its receptor, and the major effectors, phosphorylated SMADs (SMAD2/SMAD3), translocate to the nucleus to activate EMT [30–32]. Although substantial data have clarified the downstream molecular networks that trigger EMT through TGFβ, the regulatory controls of the oncogenic functions of TGFβ have not been well elucidated. In addition, the TGFβ signaling pathway exhibits a variety of biological functions, such as regulating extracellular matrix synthesis, angiogenesis, and cell line differentiation [33]. Di Guglielmo’s study demonstrated that TGFβ receptors internalize into both caveolin- and EEA1-positive vesicles and reside in both lipid raft and nonraft membrane domains. Clathrin-dependent internalization into the EEA1-positive endosomes, where the Smad2 anchor SARA is enriched, promotes the TGFβ signaling. In contrast, the lipid raft-caveolar internalization pathway involves the Smad7-Smurf2 bound receptor and is required for rapid receptor turnover [20, 34]. Therefore, the colocalization of TGFβR2 with the primary endosomal marker EEA1 or lipid raft marker Caveolin-1 was observed by confocal microscopy in our study. With downregulation of DGKZ, the colocalization of TGFβR2 with EEA1 was found to be reduced, but its colocalization with Caveolin-1 was increased. These findings suggested that the clathrin-dependent internalization of TGFβR2 was reduced, while lipid raft-caveolar internalization was increased, ultimately leading to the inhibition of the TGFβ signaling pathway.

The main components of the cell membrane include lipid molecules such as phospholipids and cholesterol. DGKZ catalyzes the conversion of DAG to PA. PA is a type of lipid molecule with unique biological functions and acts as a metabolic intermediate or the basic component of the biological membrane. PA is also a second messenger that can play a critical role in regulating some signaling processes [35, 36]. While many targets of PA signaling have been identified, the most critical target of PA in cancer cells is likely to be mTOR, the mammalian target of rapamycin [37]. A lipid signaling cascade involving phospholipase D (PLD) and PA has also been found to mediate mitogenic signaling upstream of mTORC1 [38]. A former study also confirmed a role for PA in mTOR regulation and described a novel pathway in which DGKZ-derived PA acts as a mediator of mTOR signaling [39]. Our results showed that the level of PA in cells was reduced after the knockout of DGKZ. Therefore, we hypothesized that the catalytic product PA of DGKZ might affect the structure or the fluidity of the plasma membrane, thereby altering the plasma membrane localization of TGFβR2. Additionally, we further isolated lipid rafts of the plasma membrane for protein extraction and found that the amount of TGFβR2 in the lipid rafts decreased after overexpression of DGKZ and that the decreasing trend in TGFβR2 was more significant when TNBC cells were cultured with PA, which could confirm our hypothesis.

In summary, our data showed that overexpression of DGKZ results in an increase in its metabolite PA, which may alter TGFβR2 partitioning by affecting the fluidity of the plasma membrane, thereby reducing TGFβR2 degradation, enhancing the phosphorylation of Smad3, upregulating the TGFβ signaling pathway and EMT, and thus promoting metastasis. These results indicate that DGKZ may serve as a novel promoter of TNBC. Although the specific pathway through which PA alters TGFβR2 partitioning as the metabolite of DGKZ is still unclear, our findings that DGKZ enrichment promotes invasion and metastasis, while its deficiency suppresses these processes, provide insights into mechanisms and strategies for therapeutic intervention in TNBC.

## Materials and methods

### Transwell assay

Cells (5 × 10^4^ for the migration assay and 1 × 10^5^ for the invasion assay) in serum-free medium were seeded in the top chambers of a 24-well plate containing inserts with a noncoated membrane or a Matrigel-coated membrane (BD Biosciences, Franklin Lakes, NJ, USA). Medium supplemented with 20% serum was added to the lower chamber as a chemoattractant. After incubation for 8–18 h, cells that did not migrate through the pores were removed with a cotton swab, while those invading the opposite side of the membrane were stained with methanol and 0.1% crystal violet for 30 min and were then counted.

### In vitro cell growth and wound-healing assay

In the cell growth assay, cells were seeded in 96-well plates at a concentration of 2 × 10^3^/200 μl in medium and imaged using an IncuCyte ZOOM System (Essen BioScience, Ann Arbor, Michigan, USA). Frames were imaged at 6-h intervals in four separate regions per well. Cultures were maintained at 37 °C in an incubation chamber, and the growth rate was analyzed using IncuCyte software (2013A Rev2). In the wound-healing assay, cells were seeded in 96-well plates (Essen ImageLock, Essen Instruments) at a concentration of 3 × 10^4^/100 μl in medium, and a wound was made by scratching the cell layer with a wound-making tool (Essen Instruments). Wound confluence was also monitored using the IncuCyte ZOOM System every 6 h for 2 days by comparing the mean relative wound density of at least three biological replicates.

### In vivo assay

All animal work was performed in accordance with the guidelines of the Institutional Animal Care and Use Committee of Fudan University under approved protocols. All animals were housed in a specific pathogen-free room and maintained on 12-h light/dark cycle at 25 °C ± 1 °C. All animals were randomly divided into control group and experimental group. To establish the in vivo lung metastasis xenograft model, 2 × 10^5^ cells labeled with GFP/luciferase were washed in serum-free DMEM and injected into 6-week-old female BALB/c nude mice (*N* = 6) through the tail vein in a total volume of 100 μl. To establish the in vivo mammary fat pad xenograft model, 2 × 10^6^ MDA-MB-231LM2 cells labeled with GFP/luciferase were washed in serum-free DMEM and injected directly into the mammary fat pads of 6-week-old NOD/SCID mice (*N* = 6) in a total volume of 50 μl. Tumors were measured at least once a week for the duration of the study with a digital caliper, and tumor volumes were calculated by the following equation: volume = length × (width)^2^/2. After 6–8 weeks, the mice were euthanized. Their lungs were removed, imaged (Bruker, Billerica, Massachusetts, USA), and placed into 4% paraformaldehyde for paraffin embedding, while the tumors were used for direct extraction of tissue proteins. Paraffin sections were stained with hematoxylin & eosin staining (H&E) according to standard protocols, and metastatic nodules in the lungs were counted with a microscopic count assay.

### Enzyme-linked immunosorbent assay

Cells (3 × 10^6^ for dilution and resuspending in PBS) were frozen and melted repeatedly three times to release the intracellular components, and the supernatant was collected after centrifugation at 4 °C for 30 min. The reagent was diluted to the working concentration, the sample was loaded to the bottom of the well of an enzyme-linked immunosorbent assay (ELISA) plate (Creative Diagnostics, Shirley, New York, USA), and 100 μl of the conjugate reagent was added to each well. Then the plate was incubated at 37 °C for 1 h, washed five times gently, and dried thoroughly. Chromogenic reagents A and B (both 50 μl) were added in an orderly manner to each well and mixed for 10 min at 37 °C. The gradient color development in the standard well was monitored, and a stop solution was added to each well in time to stop the reaction. After the termination of the reaction, the enzyme plate analyzer was used for the determination immediately, and the detection wavelength was set at 450 nm. The standard substance was used to draw the standard curve and calculate the sample concentration.

### Isolation of plasma membrane-derived lipid rafts

A Minute™ Plasma Membrane-Derived Lipid Raft Isolation Kit LR-042 (Beijing, China) was utilized in this part.

### Study population

This study involved 200 cases of invasive ductal breast cancer with a median follow-up of 9 months. The diagnoses were verified by two independent pathologists in the Department of Pathology of Fudan University Shanghai Cancer Center (FDUSCC, Shanghai, China). Samples were collected from these patients in the Department of Breast Surgery of FDUSCC between 2004 and 2008. Tissue microarrays (TMAs) were obtained from archived formalin-fixed, paraffin wax-embedded carcinoma samples. This study was approved by the Ethics Committee of FDUSCC, and each participant signed an informed consent document.

### Statistical analysis

All experiments were carried out at least three times in triplicate. All the statistical tests were justified as appropriate. Analysis of variance was performed and assumption criteria were met and analysis of variance was performed. Results were expressed as mean ± standard deviation (SD). Statistical analysis was performed using SPSS 22.0 software (SPSS, USA) and PRISM 8 (GraphPad Software Inc, USA). Comparisons of quantitative data between two groups were analyzed with a *t*-test (two-tailed; *p* < 0.05 was considered significant). The *χ*^2^-test was used to compare qualitative variables. The survival curves were constructed according to the Kaplan–Meier method and compared with the log-rank test. Disease-free survival was defined as the time from surgery to the first date of local recurrence, regional recurrence, distant recurrence, second breast cancer, or death from any cause, whichever occurred first. Overall survival was assessed from the date of surgery to the date of death or last follow-up. Patients without events or death were censored at the last follow-up. Univariate and multivariate analyses were based on the Cox proportional hazards regression model.

## Supplementary information

Supplementary Information

aj-checklist

## Data Availability

The data that support the findings of this study are available from the corresponding author upon reasonable request.
