# Supplementary material for: DGKZ promotes TGFβ signaling pathway and metastasis in triple-negative breast cancer by suppressing lipid raft-dependent endocytosis of TGFβR2
Source: Cell Death Dis. 2022 Feb 3;13(2):105. doi: 10.1038/s41419-022-04537-x (PMC8814002; doi:10.1038/s41419-022-04537-x)
Supplement: Supplementary file 1 — Supplementary Information [file 41419_2022_4537_MOESM1_ESM.docx]

**Supplementary Information**

**Supplementary materials and methods**

**Cell culture**

Breast cancer cell lines (MDA-MB-436, MDA-MB-453, MDA-MB-468, MDA-MB-231, BT-20, BT-549, Hs-578T, MDA-MB-231LM2, MDA-MB-231#4175) and HEK 293T cells were obtained from the Shanghai Cell Bank, Type Culture Collection Committee of Chinese Academy of Science (Shanghai, China), and they were authenticated using short tandem repeat (STR) profiling by the Cell Bank. MDA-MB-231BO cells were obtained from Dr. Toshiyuki Yoneda (The University of Texas, USA)[1, 2]. The highly metastatic cell line MDA-MB-231HM was developed from parental MDA-MB-231 cells by selection *in vivo*, authenticated and obtained a patent. The cell lines were maintained in complete growth medium as recommended by the suppliers. Liquid nitrogen stocks were created upon receipt, and these cell lines were maintained in our laboratory and subjected to routine cell line quality examinations (e.g., morphology, mycoplasma contamination) by HD Biosciences every 3 months. Cells were used for no more than 6 months after being thawed.

**Quantitative real-time PCR**

Total RNA was extracted with TRIzol reagent (Invitrogen, Carlsbad, California, USA) and reverse transcribed using a PrimeScript RT Reagent Kit (TaKaRa Biotechnology, Japan). Subsequently, real-time PCR was performed with SYBR Premix Ex Taq (TaKaRa Biotechnology, Japan) using an ABI Prism 7900 instrument (Applied Biosystems, Foster City, California, USA). The primer sequences used in this study are listed in Supplementary Table 1.

**Plasmids and small guide RNA(sgRNA)**

DGKZ cDNA for overexpression experiments using the pCDH-CMV-MCS-EF1-Puro lentiviral vector was purchased from Genewiz (South Plainfield, New Jersey, USA), as were DGKZ sgRNAs. The primer sequences are listed in Supplementary Table 1.

**Lentiviral packaging and transduction**

The 293T cells were cotransfected with lentiviral vectors and the packaging and envelope plasmids psPAX2 and pMD2G. Forty-eight hours after transfection, the viral supernatants were collected, filtered and concentrated by ultracentrifugation. Polybrene (Sigma-Aldrich, Natick, USA) was added at a working concentration of 10 ng/ml. Cells were sequentially incubated with virus for 8 hours for transduction, washed with phosphate-buffered saline (PBS), and incubated with medium containing fetal bovine serum (FBS). Thirty-six hours later, the transduced cells were subjected to selection with 2 μg/ml puromycin for 10 days. Ectopic expression was verified by western blot analysis of DGKZ.

**Western blot analysis**

Cells were lysed in Pierce T-PER (Tissue Protein Extraction Reagent; Thermo Fisher Scientific Inc., Waltham, Massachusetts, USA) containing protease inhibitor cocktail tablets and phosphatase inhibitors (Roche, Basel, Switzerland). Equal amounts of cell lysates were resolved by sodium dodecyl sulfate polyacrylamide gel electrophoresis (SDS–PAGE) and transferred to polyvinylidene fluoride (PVDF) membranes (Millipore, Burlington, Massachusetts, USA). The membranes were blocked in 5% nonfat dry milk or 5% bovine serum albumin and were then incubated with primary antibodies followed by the appropriate horseradish peroxidase (HRP)-conjugated secondary antibodies. Immunoreactive bands were detected using enhanced chemiluminescence. For quantification, the western blot films were scanned and analyzed using ImageJ Version 1.33u software (National Institutes of Health, Bethesda, Maryland, USA). The antibodies used in this study are listed in Supplementary Table 2.

**Immunofluorescence**

For immunofluorescence staining, cells were plated on cover slides, fixed with 4% paraformaldehyde, permeabilized with 0.3% Triton X-100, and blocked with 1% BSA. Cells were incubated with the primary antibodies overnight and were then incubated with the corresponding fluorescent dye-conjugated secondary antibody for labeling. The localizations were observed, and pictures were captured using a Leica TCS SP2 confocal system (Leica, Wetzlar, Germany).

**Immunohistochemical analysis**

Paraffin-embedded tissue sections were deparaffinized at 60°C for 20 min, cleared in xylene, and subjected to a graded series of alcohol. For immunohistochemical (IHC) analysis, the slides were heated with saline sodium citrate buffer at 95° to 100°C. After cooling, the slides were blocked with blocking solution (2% goat serum, 2% bovine serum albumin, and 0.05% Tween-20 in PBS) at room temperature and incubated with a primary antibody diluted in blocking solution at 4°C. Endogenous peroxidase activity was quenched with 0.3% H_2_O_2_. Slides were incubated with a HRP-conjugated secondary antibody at room temperature, developed with 3,3′-diaminobenzidine substrate (GeneTech, South San Francisco, California, USA), counterstained and blued with hematoxylin, and dehydrated with a graded series of alcohol. The positive staining density was measured using a computerized imaging system composed of a Leica DFC420 CCD camera connected to a Leica DM IRE2 microscope (Leica Microsystems Imaging Solutions Ltd., Wetzlar, Germany). The H-scoring system, which evaluates the staining intensity (0 to 3) and the percentage of positively stained cells (0 to 4) to yield a final score ranging from 0 to 12, was used. Sections with scores of 0 to 3 were considered negative, while those with scores of 4 to 12 were considered positive.

Supplementary Table 1 The primer sequences used in this study

| DGKZ-F | 5’-CGGAGGCCCCAGAATACTCT-3’ |
| --- | --- |
| DGKZ-R | 5’-TTGTCGGGGATTGAGATACCA-3’ |
| β-actin-F | 5’-CATGTACGTTGCTATCCAGGC-3’ |
| β-actin-R | 5’-CTCCTTAATGTCACGCACGAT-3’ |
| TGFβR1-F | 5’-GCTGTATTGCAGACTTAGGACTG-3’ |
| TGFβR1-R | 5’-TTTTTGTTCCCACTCTGTGGTT-3’ |
| TGFβR2-F | 5’-AAGATGACCGCTCTGACATCA-3’ |
| TGFβR2-R | 5’-CTTATAGACCTCAGCAAAGCGAC-3’ |
| TGFβ1-F | 5’-CCCTGGACACCAACTATTGC-3’ |
| TGFβ1-R | 5’-CTTCCAGCCGAGGTCCTT-3’ |
| Smad3-F | 5’-TGGACGCAGGTTCTCCAAAC-3’ |
| Smad3-R | 5’-CCGGCTCGCAGTAGGTAAC-3’ |
| Smad4-F | 5’-CTCATGTGATCTATGCCCGTC-3’ |
| Smad4-R | 5’-AGGTGATACAACTCGTTCGTAGT-3’ |
| sgRNA1-F | 5’-CACCGCCGGAGCAGCGACTCCGAGT-3’ |
| sgRNA1-R | 5’-AAACACTCGGAGTCGCTGCTCCGGC-3’ |
| sgRNA2-F | 5’-CACCGGCCTCGGGGCTACCGTCCCG-3’ |
| sgRNA2-R | 5’-AAACCGGGACGGTAGCCCCGAGGCC-3’ |

Supplementary Table 2 The antibodies used in the study

| Antibody | Dilution | Company |
| --- | --- | --- |
| Rabbit anti-E-cadherin | 1: 1000 | Proteintech Cat#20874-1-AP |
| Rabbit anti-N-cadherin | 1: 5000 | Proteintech Cat#22018-1-AP |
| Mouse anti-Fibronectin 1 | 1: 1000 | Proteintech Cat#15613-1-AP |
| Rabbit anti-Snail | 1: 1000 | Abcam Cat#ab216347 |
| Mouse anti-TGFβR2 | 1: 2000 | Proteintech Cat#66636-1-lg |
| Rabbit anti-p-Smad3 | 1: 1000 | Abcam Cat#ab52903 |
| Rabbit anti-TGFβR1 | 1: 1000 | Abcam Cat#ab235578 |
| Rabbit anti-Smad3 | 1: 1000 | Abcam Cat#ab40854 |
| Rabbit anti-Smad4 | 1: 1000 | CST Cat#46535 |
| Rabbit anti-PCNA | 1: 1000 | Abgent Cat#AP2835b |
| Rabbit anti-Flotillin 1 | 1: 20000 | Abcam Cat#ab133497 |
| Phalloidin (IF) | 1: 200 | Yeasen Cat#40774ES |
| Mouse anti-EEA1（IF） | 1: 200 | Abcam Cat#ab70521 |
| Rabbit anti-TGFβR2（IF） | 1: 200 | Abcam Cat#ab270440 |
| Mouse anti-TGFβR2（IF） | 1: 50 | Abcam Cat#ab78419 |
| Rabbit anti-Caveolin-1（IF） | 1: 500 | Abcam Cat#ab32577 |
| Secondary antibody for IF | 1: 500 | Abcam Cat#ab185043 |
| Rabbit anti-DGKZ | 1: 1000 | Abcam Cat#ab239080 |
| Goat anti-rabbit IgG | 1: 5000 | SAB Cat#L3012 |
| Goat anti-mouse IgG | 1: 5000 | SAB Cat#L3032 |
| HRP-conjugated Mouse GAPDH | 1: 20000 | Proteintech Cat#HRP-60004 |

**
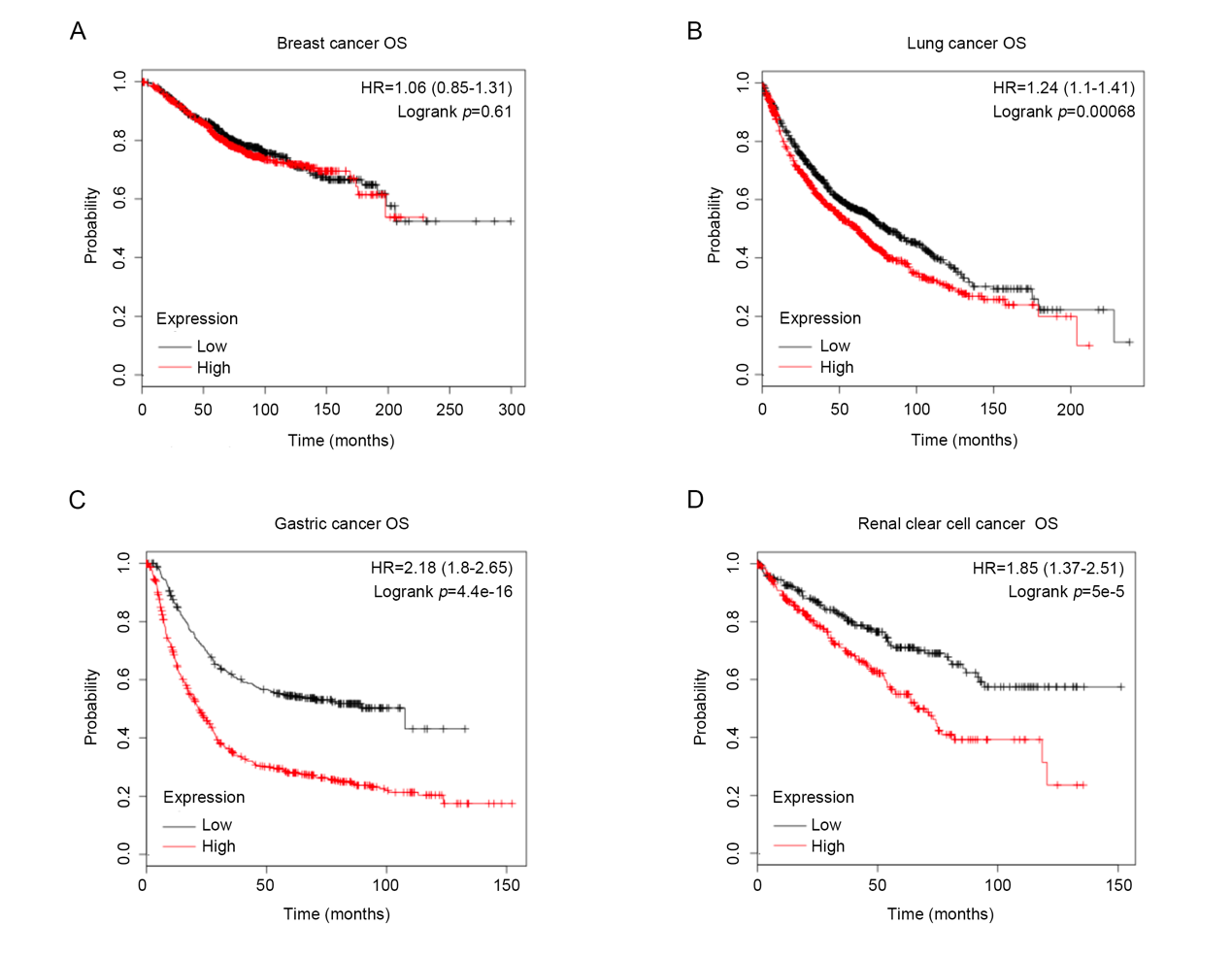
**

**Supplementary Fig. 1** The correlation of overall survival (OS) of breast cancer, lung cancer, gastric cancer and renal clear cell cancer patients with the expression of DGKZ

**
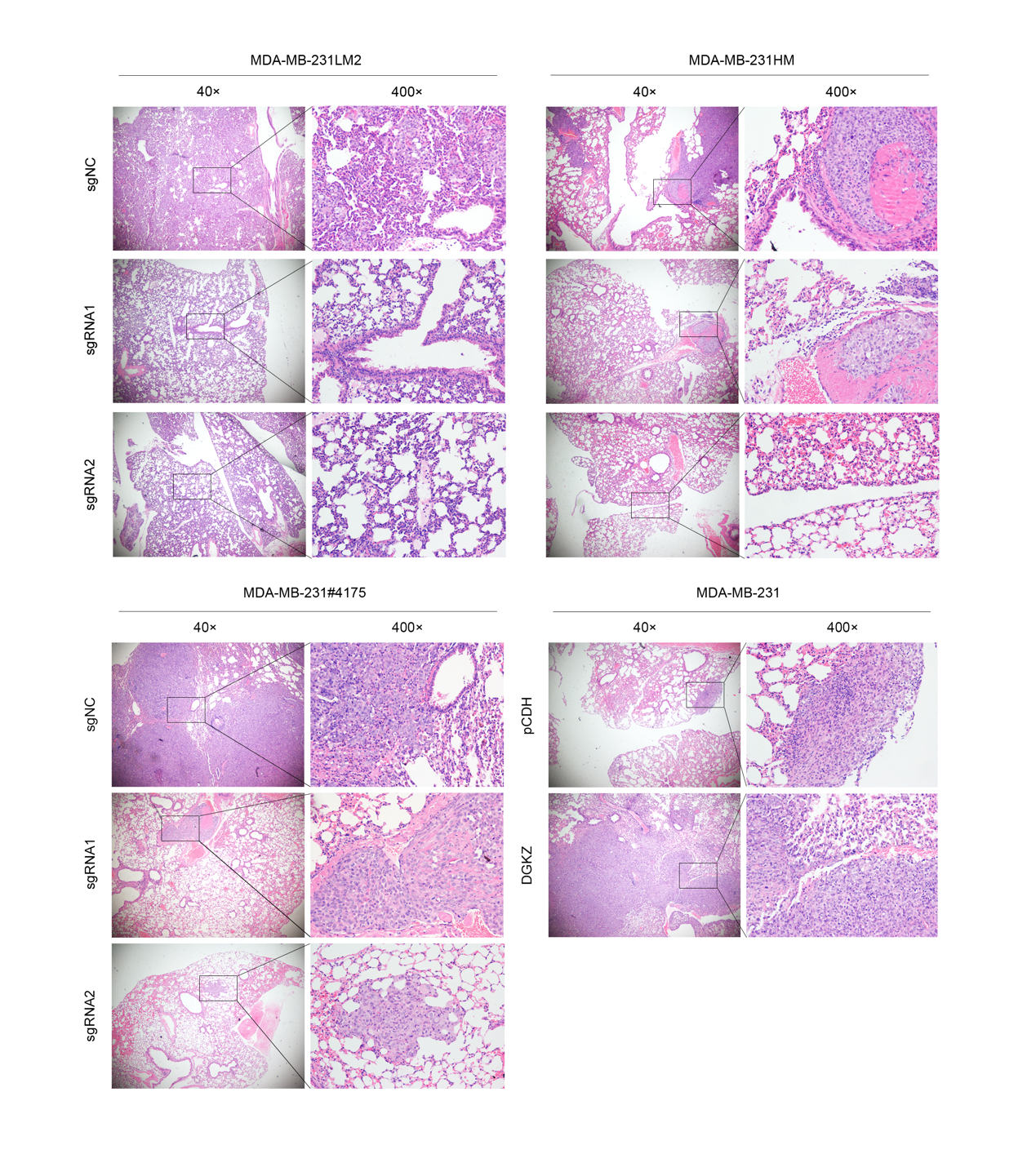
**

**Supplementary Fig. 2** HE staining of lung tissue revealed that the downregulation of DGKZ significantly decreased lung metastasis and lung metastasis was elevated after DGKZ was upregulated.


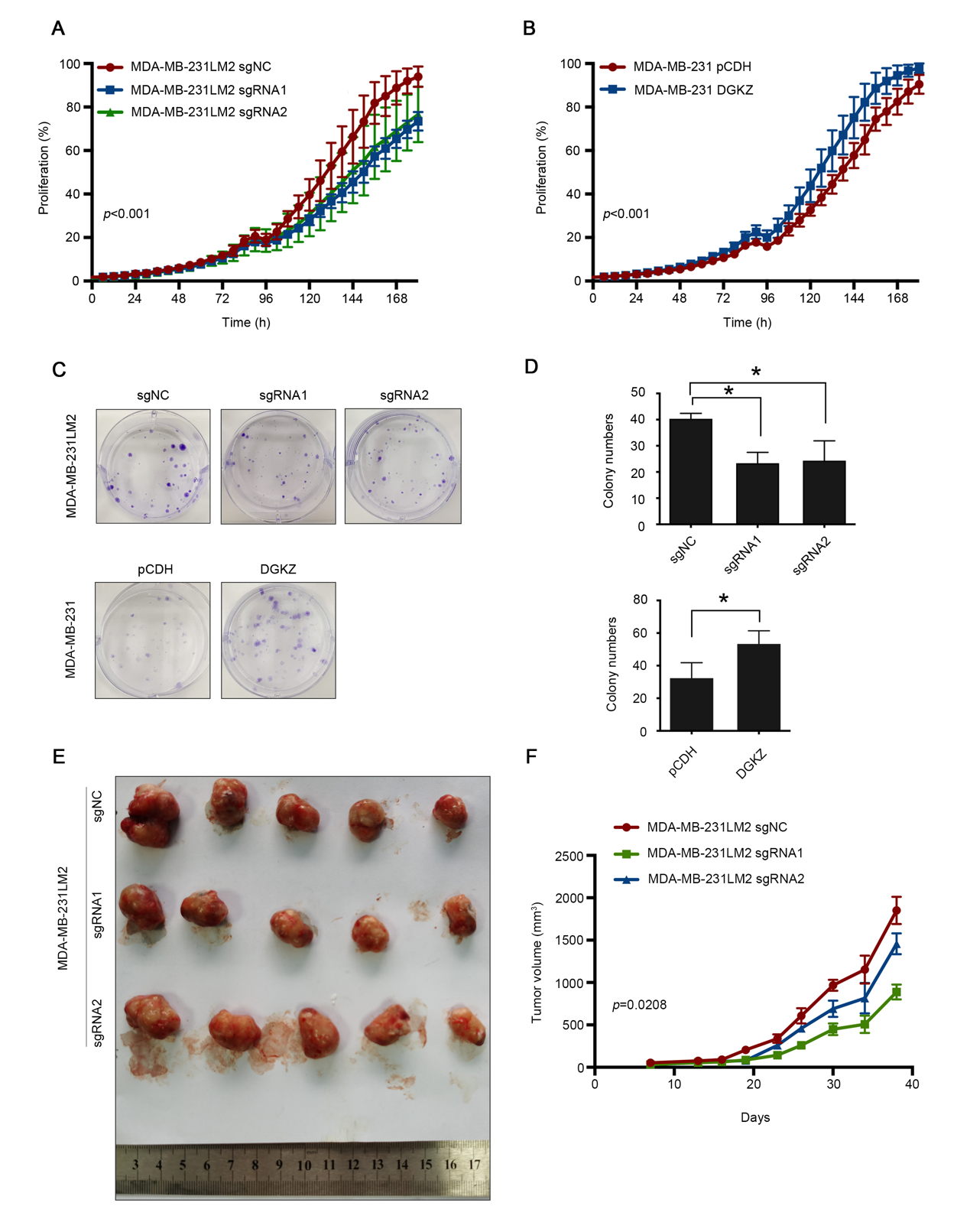
**Supplementary Fig.**
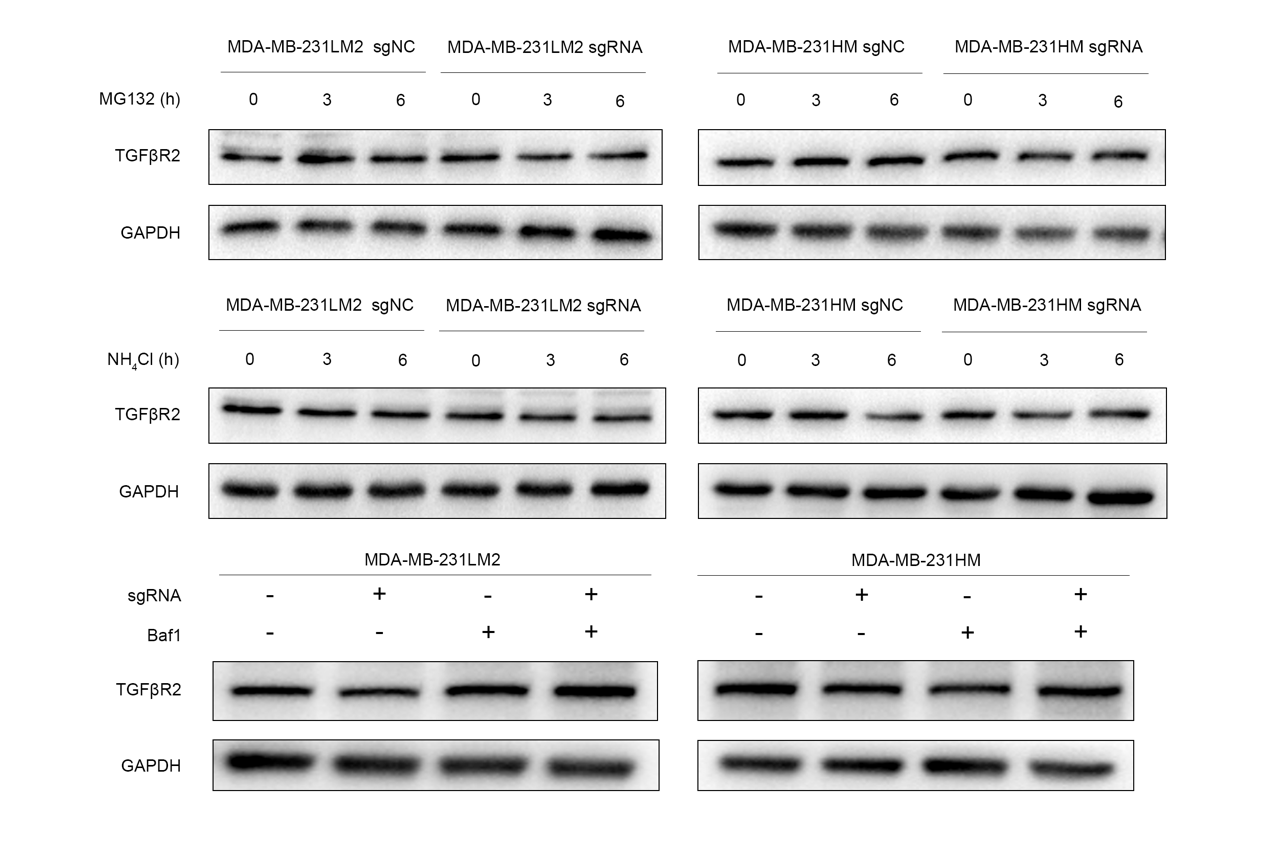
**3** (A) The alteration of proliferation ability of 231LM2 cells after the knockout of DGKZ. (B) The alteration of proliferation ability of 231 cells after the overexpression of DGKZ. (C) The alteration of clone formation ability of the above cells. D: Statistical graph of the number of clones in C. (E) Tumor tissues in situ were removed from mice after death at the end of the experiment (removed the outliers, which were too far from the 25th percentile or the 75th percentile of the box plot). (F) Tumor volume of mice was recorded at least once a week and statistically mapped. Bars, ±SD; * *p*<0.05.

**
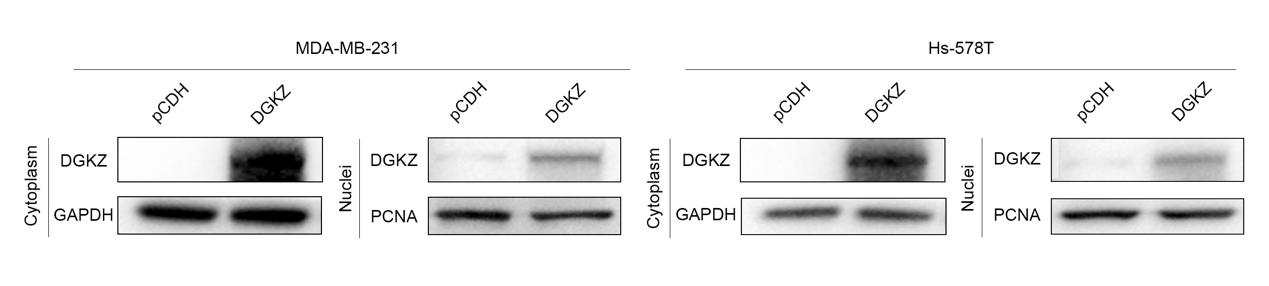
**

**Supplementary Fig. 4** Western blot analysis of DGKZ-overexpressed MDA-MB-231 and HS578-T cells by the nuclear and cytoplasmic separating extraction assay to detect the subcellular localization of DGKZ.

**
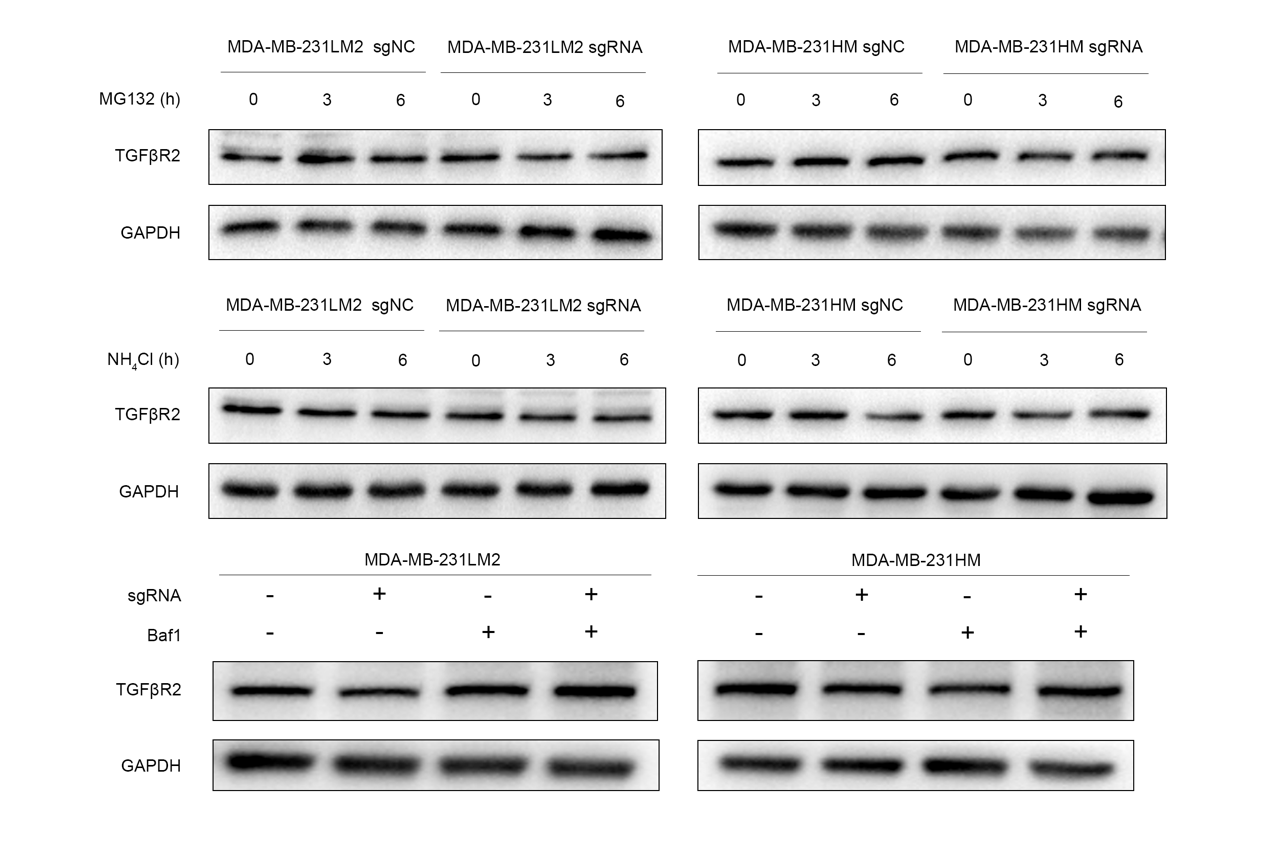
**

**Supplementary Fig. 5** Western blot analysis of the expression of TGFβR2 in the DGKZ-knockout cells treated with MG132 (a ubiquitin proteasome inhibitor), NH4CL (a lysosomal pathway inhibitor) and bafilomycin A1 (a lysosomal pathway inhibitor, BAF1).

**Reference**

1. Stark AM, Anuszkiewicz B, Mentlein R, Yoneda T, Mehdorn HM, Held-Feindt J. Differential expression of matrix metalloproteinases in brain- and bone-seeking clones of metastatic MDA-MB-231 breast cancer cells. J Neurooncol. 2007;81:39-48.

2. Hiraga T, Kizaka-Kondoh S, Hirota K, Hiraoka M, Yoneda T. Hypoxia and hypoxia-inducible factor-1 expression enhance osteolytic bone metastases of breast cancer. Cancer Res. 2007;67:4157-63.
